# Retrospective Analysis of the Therapeutic Outcomes of Microneedle Radiofrequency on Melasma by Optical Coherence Tomography: A Observational Pilot Study

**DOI:** 10.3390/diagnostics16131957

**Published:** 2026-06-24

**Authors:** Yi-Teng Hung, Feng-Ling Tsai, Yau-Li Huang, Chih-Wei Lu, Hsing Cheng, Chien-Ming Chen

**Affiliations:** 1 Belléesse Dermatologic Clinic, Taipei 220, Taiwan; herovincent25@hotmail.com.tw; 2 Sunshine Dermatologic Clinic, New Taipei City 244, Taiwan; 3 Apollo Medical Optics, Ltd., Taipei 114, Taiwan; emilyee@gmail.com (F.-L.T.); cwlu323@gmail.com (C.-W.L.); chengmailvivian09@gmail.com (H.C.); 4 Department of Dermatology, Chang Gung Memorial Hospital, Linkou, Taoyuan 333, Taiwan; henryhuang0219@gmail.com; 5 College of Medicine, Chang Gung University, Taoyuan 333, Taiwan; 6 Department of Cosmetic Science, Chang Gung University of Science and Technology, Taoyuan 333, Taiwan

**Keywords:** melasma, microneedle radiofrequency, optical coherence tomography, pigmentary disorder

## Abstract

**Background**: No preferred treatments for melasma are known, owing to its underlying complicated pathomechanisms; microneedle radiofrequency (MRF) has recently been used to treat melasma. **Objectives**: We aimed to investigate the effects and pathomechanisms of melasma treated by MRF and identify the possible determining factors for good response. **Methods**: Therapeutic outcomes were measured using the Melasma Area and Severity Index (MASI) and artificial intelligence-assisted optical coherence tomography (OCT) evaluation for collagen and pigmentation at baseline and 2 months after each treatment. Participants were divided into good- (≥25% reduction in MASI) and poor-response (<25% reduction in MASI) groups after the last MRF treatment. **Results**: Two patients achieved fair response and three patients achieved poor response. Overall OCT analysis showed that the confetti/granular melanin ratios (melanin aggregation index) decreased, the distance between melanosomes increased, and the size of melanin decreased. The number of dendritic cells (DCs) decreased. In subgroup analysis, the continuity of the basement membrane was improved in the fair-response group, and the melanin aggregation index and the number of DCs were decreased in the poor-response group. A higher baseline confetti/granular melanin ratio trended towards poorer therapeutic response. **Conclusions**: This pilot study used OCT to assess the therapeutic efficacy of MRF for melasma and identify the characteristics of individuals for whom MRF is effective. The statistical results were exploratory and descriptive. Further large-scale, randomized controlled studies are required to prove the efficacy of MRF in treating melasma and the feasibility of OCT in investigating the treatment response of melasma.

## 1. Introduction

Melasma, also known as chloasma, is a common, easily relapsing, facial pigmented disorder that often affects females and darker skin types [1,2,3]. The etiologies of melasma are complicated and diverse, including ultraviolet radiation, sex hormonal influences, inflammatory processes, epidermal–dermal microenvironments, and genetic factors [1,2,3,4]. The treatment of melasma is challenging due to its high recurrence rates and postinflammatory hyperpigmentation. Although broad-spectrum sunscreens, topical/systemic lightening agents, chemical peeling, laser and light devices, and microneedling have been used to treat melasma with some clinical results, no single treatment has been universally effective [1,2,3,4].

Therefore, more melasma treatments still need to be investigated. Microneedle radiofrequency (MRF) is a minimally invasive treatment that transmits radiofrequency-generated thermal energy to the dermo-epidermal junction and dermis to stimulate neocollagenesis by producing tiny perforations and minimal ablation in the epidermis [4,5,6]. The device delivers an accurate amount of energy into the adjustable depth according to the specific therapeutic target and decreases the risks of postinflammatory pigmentation or scarring in comparison with resurfacing laser and peelings [7]. MRF has been strongly recommended for and used in the treatment of skin rejuvenation, acne vulgaris, acne scars, surgical scars, and striae [6]. MRF can remodel the dermal extracellular matrix, stabilize the basement membrane, and reduce inflammation, angiogenesis, and mast cell populations [4,5,8,9]. In addition, the skin punctures induced by microneedling enhance transcutaneous absorption of topical lightening products with deeper and more even placement and allow for the transcutaneous elimination of melanin [5,9]. However, so far, only two studies have reported on the effects of MRF on melasma, reporting the clinical benefits of including it in low-fluence Q-switched Nd:YAG lasers for the management of melasma [4,5]. Consequently, more studies are needed to evaluate the therapeutic effect of MRF in melasma.

Most of the studies published assess the efficacy of melasma treatments using subjective methods, such as the Melasma Area and Severity Index (MASI), the modified MASI, Patient Global Assessment, the melasma severity scale, and the melasma severity index [2,10]. Few studies have adopted objective methods, including spectrophotometry (calorimeter and mexameter), the skin hyperpigmentation index (with a smartphone dermatoscope adapter and an online calculator), and histopathology [2,9,10,11]. Recently, optical coherence tomography (OCT) has been increasingly used in assessing the efficacy of esthetic skin treatment, including melasma [12,13,14,15,16,17]. OCT was approved for dermatological use by the Food and Drug Administration (FDA) and European Medicines Agency (EMA) [18,19]. OCT is a noninvasive optical three-dimensional imaging technique that uses near-infrared as a form of coherent light interference to visualize skin microstructures beneath the surface of the skin; it can access microstructures more deeply than conventional or confocal microscopy can [12,13,16]. OCT has the following advantages: it easily recognizes epidermal and papillary dermal structures, has a large penetration depth and field of view, and has the unique ability to image vasculature, collagen, and inflammatory responses [16].

Up until now, the most effective treatment for melasma has remained uncertain, and relapses are frequent; thus, identifying the efficacy of MRF as a novel treatment for melasma is beneficial to physicians and patients. Moreover, the application of an OCT system to assess the progress and outcomes of MRF in melasma allows for objectivity and precision, and it may help in identifying the characteristics of individuals for whom MRF is effective.

## 2. Materials and Methods

### 2.1. Ethical Approval

This study was approved by the Chang Gung Medical Foundation Institutional Review Board (IRB No. 202501102B0) on 2 September 2025. Signed informed consent was obtained from the participants, who agreed to the use of their retrospective data for publication, and identifiable personal medical information was removed.

### 2.2. Study Design and Participants

In this pilot study, we enrolled five individuals diagnosed with melasma who underwent MRF on the whole face (except the orbital region and lips) at the Belléesse clinic and Chang Gung Memorial Hospital, Linkou Branch, between October 2023 and October 2024. All of the patients in this study had mixed melasma. Clinical appearance included a combination of blue–gray, light-brown ill-defined patches and scattered dark-brown, well-defined maculopatches. Melasma was determined by dermoscopic examination, including reticular networks, dark granules and globules, and/or telangiectasia. None of the participants had received additional skin-lightening treatments (including lasers, mesotherapy, and bleaching creams), took oral contraceptives within one year prior to the study, or had any significant medical history. Sunscreen use was requested for individuals during the study period. The microneedling system (Sylfirm X RF Microneedling system, Roseville, NSW, Australia) was applied with the settings of the pulsed-wave (PW) 2 model, depths of 0.6 mm and 0.3 mm, each shot overlapping 10–20%, and a power (energy) of level 4, with the endpoint being “mild” skin erythema. Sylfirm X RF combines microneedling with RF energy and offers both continuous-wave (CW) and pulse-wave (PW) RF modes. This dual-mode technology allows for skin rejuvenation (CW) and the treatment of pigmentation and vascular issues (PWs). The number of total shots per session was approximately 350 but varied between different sizes of the facial skin surface. The operating procedure was as follows: one pass with a 0.3 mm depth on the whole face and 2–3 passes with a 0.6 mm depth on the pigmentation areas (either 2 passes with mild skin erythema or 3 passes) in intervals of 8 weeks. Topical anesthesia was used before treatment and topical betamethasone (17-valerate)-gentamicin (as sulfate) cream was applied immediately after treatment.

### 2.3. Measures and Outcomes

Treatment efficacy was evaluated at baseline and 2 months after each treatment. The measured outcomes included the MASI score and OCT assessment of the left malar area at each follow-up. The MASI score was independently calculated by two dermatologists, who were blinded to the timepoint when scoring the MASI. The patients were further divided into fair response (≥25% reduction in MASI score of the left malar area) and poor response (<25% reduction in MASI score of the left malar area) based on the improvement in MASI scores 2 months after the last MRF treatment compared with those at baseline. We used a hole punching ruler and ruler stickers to locate the point of OCT examination on the same participant during each follow up. Ruler stickers were placed on both the melasma lesions and the perilesional skin. Clinical photographs were taken with and without the stickers at each visit. During follow-up visits, the ruler stickers were positioned at the locations corresponding to those used in previous visits and confirmed by the hole punching ruler to ensure that OCT assessments were performed consistently on the same sites. The OCT results in the fair- and poor-response groups were analyzed and compared. A confetti/granular melanin ratio, as the melanin aggregation index, was assessed by OCT and calculated to determine the threshold of poor treatment efficacy.

### 2.4. OCT Acquisition

The methods and technical specifications of the instruments (ApolloVue S100, Apollo Medical Optics, Ltd., Taipei, Taiwan), as well as the approaches for analyzing collagen, melanosomes, and dendritic cells, have been comprehensively detailed in previous studies. The lateral resolution is below 1 μm, and the experimentally measured axial resolution is 1.32 μm [20].

The OCT instrument continuously acquired en face slices with a step size of 0.56 µm from the interference point (skin surface reference) to a depth of 400 µm. This continuous acquisition allowed for the visualization of skin microstructures at specific depths within the epidermal and dermal layers. Cross-sectional OCT images were obtained as previously described [21].

### 2.5. OCT Assessment

For each en face continuous scan (C-scan) stack, three corresponding cross-sectional images were obtained. The dermo-epidermal junction was identified based on the textural contrast between the epidermal and dermal structures. The overall depth of the dermo-epidermal junction for the en face stack was then defined as the top 2% of the detected dermo-epidermal junction depths across the cross-sectional images.

For cross-sectional images, the dermo-epidermal junction was identified based on the textural contrast between the epidermal and dermal structures.

A computer-aided detection system [13,14,22] was used to extract statistical data on skin characteristics, including melanin, dendritic cells, and collagen. “Confetti melanin” and “granular melanin” are OCT markers for assessing skin pigmentation. Targets with a diameter >0.5 µm and a brightness level >153 in gray scale were defined as “detected” melanin [22,23]. Artificial intelligence (AI) automatically detected and determined the following two types of melanin: “confetti” (yellow mark) with a pigment particle diameter >3.3 µm and an area >8.427 µm^2^ and “granular (red mark) with a pigment particle diameter of 0.5–3.3 µm and an area of 0.28–8.427 µm^2^. The melanin aggregation index was defined as the ratio of confetti to granules. If the aggregation index before treatment is high, then melanin becomes increasingly aggregated. A visual inspection was conducted to evaluate basement membrane (BM) disruption. The “Ruler” tool in AMO’s Explorer was employed to measure the length of the disrupted BM. Hyporeflective long linear features observed between the epidermis and dermis corresponded to periodic acid–Schiff-stained sections described in previous studies [24]. In disrupted regions, the border between the epidermis and dermis remained visible; however, the characteristic “black line” was absent, consistent with the blurred BM features reported in earlier research. A rule-based (AI) system was employed to evaluate collagen remodeling in OCT images using a structured three-phase pipeline. The algorithm was trained on a database comprising 114 cross-sectional OCT images of facial skin obtained from healthy volunteers with a cellular resolution of more than 200. Optical signals corresponding to collagen fibers were quantified and analyzed using the Frangi vesselness method [22]. The resulting collagen grade ranged from 0 to 100, where scores of 0–39 corresponded to aging skin, 40–70 to an intermediate skin condition, and 71–100 to healthy skin. The final collagen grade was generated by a deep learning model incorporating collagen fiber-related parameters, including collagen fiber length, fiber area, collagen density, fragmentation, brightness, and other related parameters. The accuracy of the model was 93.3%; it was validated against human expert scoring for each cross-sectional image. The optical signals corresponding to collagen fiber were detected and quantified in vertical OCT images (B-scan). Roundness is a quantitative parameter used to assess the shape of collagen fiber structures. It measures how close an object is to a perfect circle, with values ranging from 0 to 1. Values closer to 1 indicate a more circular shape, which reflects a higher degree of collagen fragmentation. Expert evaluators reviewed the images and classified them into five categories. The first category represented the highest ratio of linear collagen to short fragmented collagen, while the fifth category represented the lowest ratio. These categories corresponded to healthy skin, an intermediate skin condition, and aging skin. Nine categories were described, as there were intermediate ratings within each. The same set of images was subsequently analyzed using the collagen detection model. The model-generated collagen health grade (ranging from 0 to 100) was compared with expert classifications. Five categories were linearly rescaled to a 0–100 scale for interpretability using the formula (−20x) + 110. For AI training, features such as area, density, size, roundness, and length of the detected collagen structures were extracted from the OCT images. A Quadratic Support Vector Machine model was used for classification, achieving a root mean square error of 0.784, indicating a robust correlation between AI-derived collagen features and expert annotations.

### 2.6. AI-Assisted Analysis

For AI-assisted analysis applied to collagen fiber quantification, we validated, against expert evaluation, that collagen analysis within the selected observation depth (approximately 100 µm below the dermo-epidermal junction) demonstrated stable OCT signal characteristics suitable for quantitative assessment. Because skin affected by melasma may exhibit heterogeneous scattering properties, an image quality control step was incorporated prior to AI analysis. Images with substantial artifacts or segmentation-interfering features, including motion blur, hair follicles, hair, dilated blood vessels and oil bubbles, were excluded from quantitative analysis.

Due to the high-resolution nature of cellular-level OCT imaging, image-quality filtering was necessary to ensure reliable segmentation. Approximately 31.8% of the acquired images were excluded during this quality control process. Image review at the quality evaluation step minimized segmentation-related errors. Following clinician review based on predefined image-quality criteria, only OCT images meeting the predefined image-quality criteria were included for AI-based detection and quantitative analysis. The representative examples with AI-generated overlays and corresponding clinician review are provided (Supplementary Figure S1).

### 2.7. Statistical Analysis

Confetti and granular melanin were detected, and the following parameters were quantified: melanin, confetti and granular area, density, distance, melanin and confetti size, confetti/granular melanin ratio, confetti/all melanin ratio, and granular/all melanin ratio. The averages at baseline and the last follow-up visit were calculated, the *p* value was calculated, and statistical significance was assessed using Student’s *t*-test in Microsoft Excel 365. Due to the small sample size. The *p*-values were descriptive rather than confirmatory. Statistical analyses were performed in the same manner for dendritic cells, basement membrane, epidermal thickness, collagen grade, length, density, and roundness. To analyze the fair and poor-response treatment groups, the same statistical approach was applied to each group separately. Potential values for the cutoff points for the confetti/granular melanin ratio were selected from 1 to 300. Predictive performance was assessed by analyzing the receiver operating characteristic (ROC) curve and area under the curve (AUC). The optimal cutoff point was identified using the Youden index.

## 3. Results

**Figure 1:**
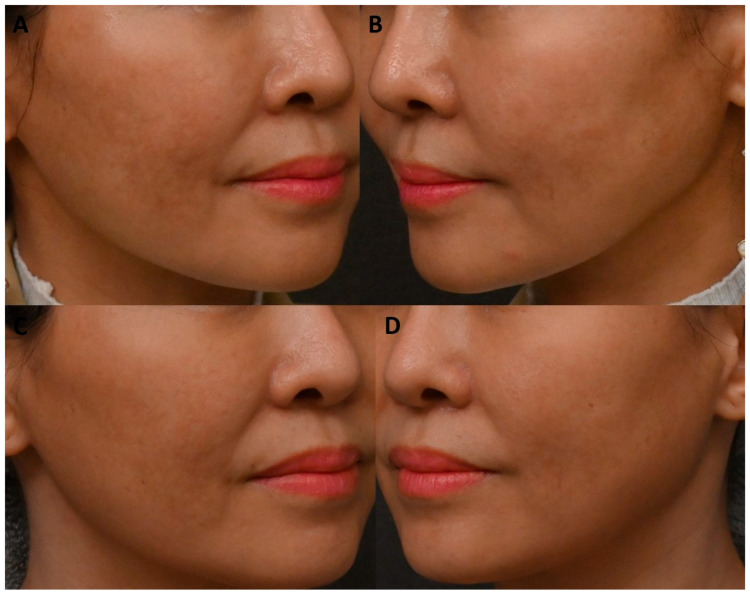
Clinical images of Patient 3 before (**A**,**B**) and after the treatment (**C**,**D**).

**Figure 2:**
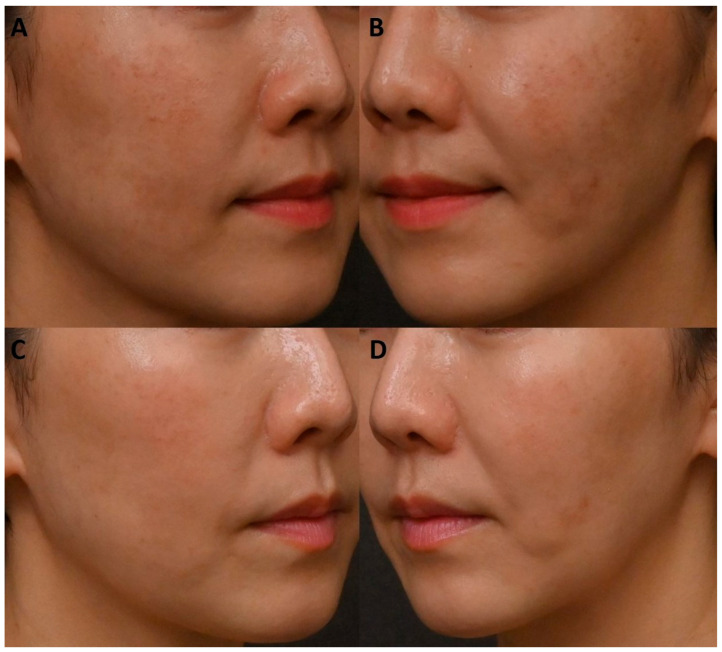
Clinical pictures of Patient 5 before (**A**,**B**) and after the treatment (**C**,**D**).

**Figure 3:**
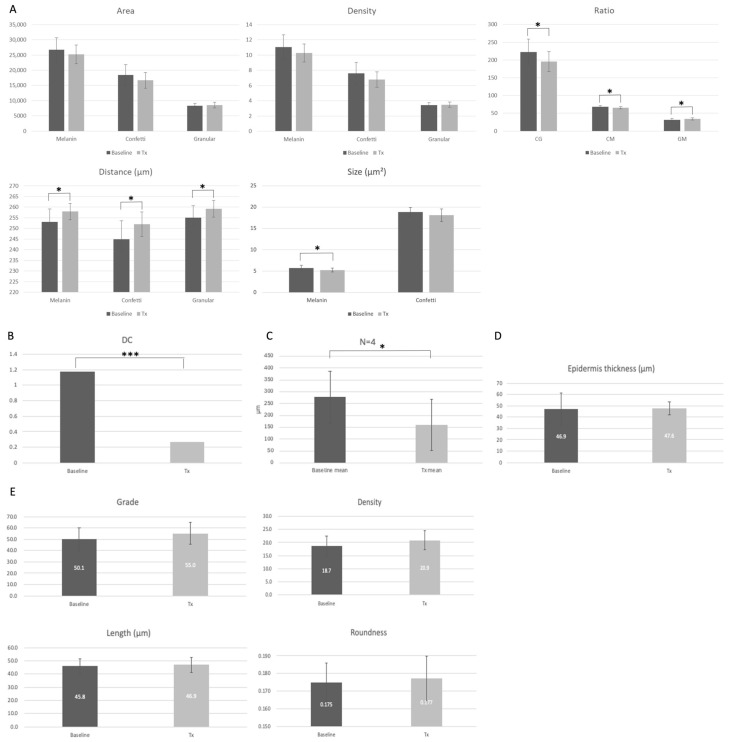
(**A**) Levels and characteristics of total melanin, confetti melanin, and confetti melanin before and after treatment. CM, confetti melanin ratio; CG, confetti granular ratio; GM, granular melanin ratio; Tx, treatment * *p* < 0.05. (**B**) The number of dendritic cells before and after treatment. Tx, treatment *** *p* < 0.005. (**C**) Disrupted area of the basement membrane before and after treatment. Patient 2 was excluded from the total number of cases because of the healthy basement membrane at baseline. N, number; Tx, treatment * *p* < 0.05. (**D**) Epidermal thickness before and after treatment. Tx, treatment. (**E**) Collagen characteristics before and after treatment. Tx, treatment.

**Figure 4:**
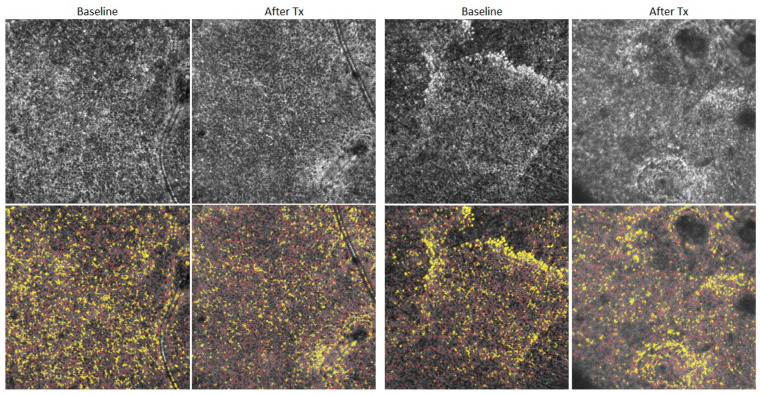
Optical coherence tomography (OCT) analysis of facial melasma (melanosome). En face OCT image of the melasma at baseline (**left**). The OCT images about 5 µm above the dermo-epidermal junction of lesional skin were analyzed. Confetti (yellow) and granular (red) melanins were detected and quantified. En face OCT image of melasma 2 months after treatment (**right**).

**Figure 5:**
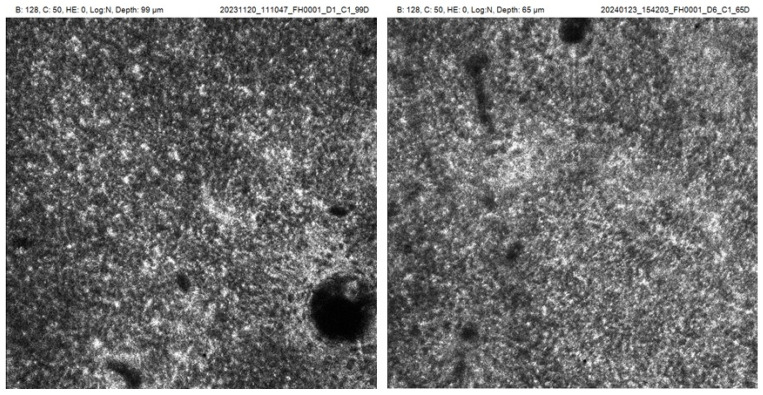
Optical coherence tomography (OCT) analysis of facial melasma (dendritic cell). En face OCT image of the melasma at baseline (**left**). The OCT image was taken 1 µm below the dermo-epidermal junction of lesional skin. Starburst and publisher-like dendritic cells were observed. En face OCT image of melasma 2 months after treatment (**right**). Starburst- and publisher-like dendritic cells were also markedly reduced.

**Figure 6:**
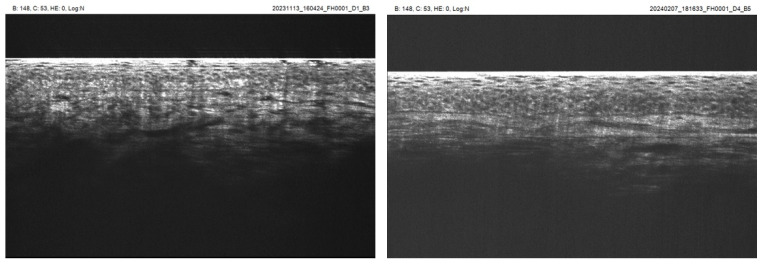
Optical coherence tomography (OCT) analysis of facial melasma (basement membrane). Cross-sectional OCT image of melasma at baseline (**left** panel). The basement membrane was disrupted, as no clear dark line was observed at the dermo-epidermal junction. Cross-sectional OCT image of melasma 2 months after treatment (**right**). The basement membrane was repaired as a dark line at the dermo-epidermal junction.

The participants included four females and one male (mean age, 50.4 [41–55] years) (Table 1). Patient 3 belongs to Fitzpatrick skin type IV, and the others are Fitzpatrick skin type III. The averaged MASI scores of each participant at baseline and at the last follow-up after Sylfirm X MRF treatment were as follows: 8.7–7.5 (Patient 1), 7.2–7.8 (Patient 2), 8.4–5.4 (Patient 3) (Figure 1), 3.6–3 (Patient 4), and 11.9–4.7 (Patient 5) (Figure 2) (Supplementary Table S1). After treatment with Sylfirm X MRF, OCT analysis showed that the average area and density of total melanin and confetti melanin decreased, the area and density of granular melanin increased, the confetti/granular, confetti/melanin, and granular/melanin ratios decreased (*p* < 0.05), the distance between melanosomes increased (the distribution of melanosomes became sparser) (*p* < 0.05), and the size of melanin decreased (*p* < 0.05) (Figure 3A and Figure 4). The average ratios of confetti/granular melanin at baseline in Patient 1, 2, 3, 4, and 5 were 234.1%, 235.6%, 215.4%, 250.4%, and 173.0%, respectively. The number of dendritic cells (DCs) decreased (*p* < 0.005) (Figure 3B and Figure 5). The disruption area of BM decreased after MRF treatment (277.4 ±154.9 μm to 160.9 ±108.8 μm, [mean ± SD], *p* < 0.05) after excluding the patient who did not have serious BM disruption at baseline (average area 30 μm, considered as healthy BM) (Figure 3C and Figure 6). The epidermal thickness (Figure 3D), collagen health grade, density, length, and roundness of the collagen (Figure 3E) were not different after treatment.

**Figure 7:**
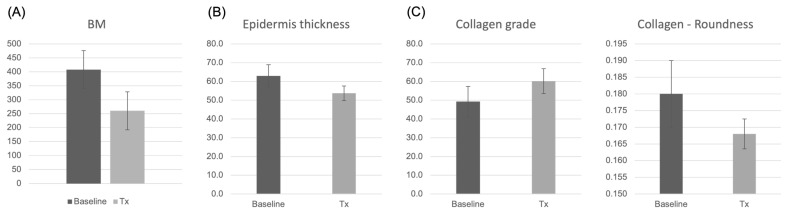
Significant changes revealed in optical coherence tomography (OCT) analysis of the fair-response group after treatment, including the disrupted area of the basement membrane (**A**); the epidermis thickness, which significantly decreased after treatment (**B**); and the health grade and roundness of collagen (**C**). BM, basement membrane; Tx, treatment.

**Figure 8:**
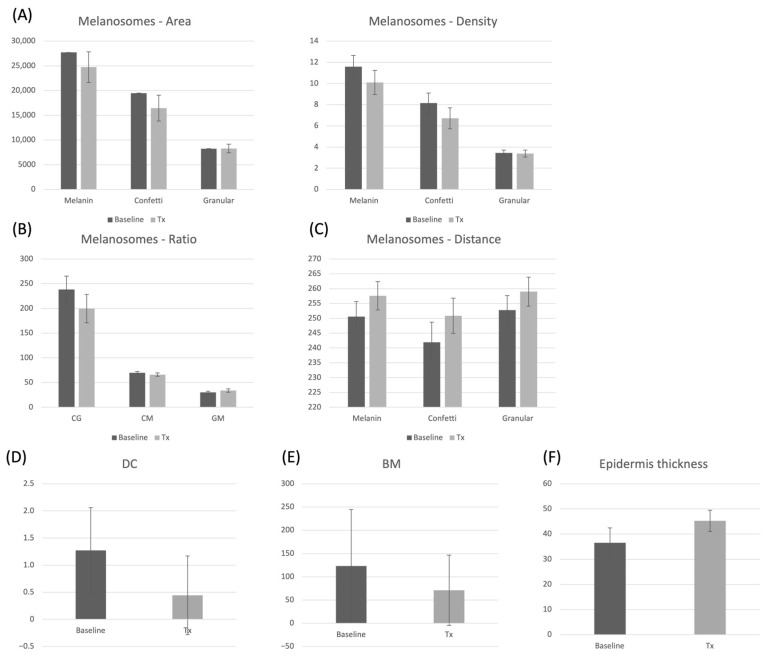
Findings from optical coherence tomography (OCT) analysis in the poor-response group, including the area and density of total melanin and confetti melanin, which significantly decreased after treatment (**A**), the ratio of confetti/granular, confetti/melanin, and granular/melanin (**B**), the distance between melanosomes (**C**), the amount of dendritic cells (**D**), the disrupted area of the basement membrane (**E**), and the epidermis thickness (**F**). BM, basement membrane; CM, confetti melanin ratio; CG, confetti granular ratio; DC, dendritic cell; GM, granular melanin ratio; Tx, treatment.

Patients 3 and 5 achieved 38% and 59% reductions in the MASI score 2 months after the last MRF treatment [fair-response group (≥25% reduction in MASI score)] and patients 1, 2, and 4 achieved 0% reductions in the MASI score [poor-response group (<25% reduction in MASI score)] (Table 1). An ROC curve was run to determine the threshold of 196%, which was associated with poor treatment response, and the AUC value was exploratory, at 0.85. The ROC analysis was conducted only for demonstration purposes and the results of it suggested the association of high confetti/granular ratio with poor treatment efficacy. However, the identified cutoff was exploratory and intended only as a preliminary signal rather than a clinically actionable threshold. The cutoff value would likely shift in larger and independent validation datasets. External validation in larger cohorts will be necessary before clinical interpretation or implementation. In the fair-efficacy group, the features of the melanosomes were not different after treatment. The number of DCs remained unchanged after the treatment. Patient 5 had many starburst DCs at baseline, but their activity decreased after treatment. The disrupted area of the BM decreased after treatment (Figure 7A). The epidermal thickness decreased after treatment (Figure 7B). The health grade and roundness of the collagen increased after treatment (Figure 7C). The collagen density and length did not differ after treatment. In the poor-response group, the area and density of total melanin and confetti melanin decreased after treatment (Figure 8A). The confetti/granular, confetti/melanin, and granular/melanin ratios decreased after treatment (Figure 8B). The distance between the melanosomes increased after treatment (Figure 8C). The number of DCs decreased after treatment (Figure 8D). The disrupted area of BM was not different after treatment (Figure 8E). The epidermal thickness increased after treatment (Figure 8F). The collagen health grade, density, length, and roundness were not different after treatment. Overall, BM and collagen grades improved in the fair-response group; melanosome and epidermis thickness improved in the poor-response group, although clinical results have not yet been shown. Last but not least, the group-level *p*-values in this pilot study were exploratory rather than predictive. The plots of individual patients’ trajectories are provided (Supplementary Table S2 and Supplementary Figure S2). Post-inflammatory erythema disappeared within a day in all the participants and no post-inflammatory hyperpigmentation event was reported thorough the study period.

## 4. Discussion

In this study, we evaluated the therapeutic effects of MRF on melasma and aimed to predict the characteristics of individuals with a good response to MRF using OCT. Two patients achieved fair response and three patients showed poor response in clinical appearance (reduction in the MASI score) 2 months after the last course of MRF treatment. The continuity of BM improved in the fair-response group, and the degree of melanin stubbornness and number of DCs decreased in the poor-response group after treatment. The results of this study were exploratory and hypothesis-generating, and larger prospective cohort studies are necessary to validate the role of OCT as a feasible tool to objectively analyze therapeutic response of melasma by MRF.

Investigating the role of each element in melasma is beneficial for promoting clearance and decreasing relapses. Currently, the complex and multi-pathophysiological conditions of melasma include pendulous melanocytes, photoaging (solar elastosis), disrupted BM, neovascularization, and increased mast cell count [25,26]. In addition to melanocytes, dermal elements contribute to the development of melasma, emphasizing the utilization of MRF and OCT to target levels on or below the BM in melasma. The basement membrane is disrupted in a total of 96% of melasma lesions, resulting in the migration of melanocytes into the dermis [3,26]. In a previous study using immunofluorescence staining, MRF was shown to repair BM disruption by significantly increasing the expression of collagen type IV in the BM [27]. The continuation of the BM was significantly increased after MRF in the fair-response group in the present study. Healthy BM can reduce both the release of melanin into the dermis and the passage of dermal melanogenic cytokines to melanocytes, thus minimizing constant and/or rebound hyperpigmentation in melasma. The addition of MRF to laser toning therapy has been proven to loosen adjacent melanocytes and keratinocytes, and melanin can be eliminated through loosened spaces [4]. Photochromophores also possess a greater capacity to absorb radiation after MRF treatment due to more widely distributed melanin [11]. Our study further concluded that the distances between melanosomes were significantly increased. MRF devices have the advantage of producing heat precisely in the dermal target tissue with minimal epidermal thermal damage and enhancing transepidermal drug delivery by electroporation to achieve dermal extracellular matrix protein remodeling and basement membrane stabilization [9]. The above findings were identified in the fair-response group of our study. Taken together, an increasing number of studies have emphasized the role of MRF in treating melasma, including synergistic treatment in combination with laser toning [4,5], monotherapy in a population with darker skin types or rebound hyperpigmentation (our study), or maintenance therapy to prevent relapse after cessation of the conventional treatments [28]. Moreover, no postinflammatory hyperpigmentation, easily occurring in laser energy treatment, was observed in our study.

OCT is a noninvasive, real-time, comprehensive, and in vivo tool for diagnosing and tracking the therapeutic responses of pigmented disorders with cellular-level resolution [14]. OCT devices offer several advantages. The imaging depth was extended to the dermal layer, offering transverse- and vertical-section plane images. Although no significant improvement was noted in the clinical assessment (reduction in MASI) in some individuals, OCT examination showed a decrease in the distribution and amount of melanin, melanosomes, and DCs. The decrease in melanin (area and density) after treatment in the poor efficacy group was likely due to severe hyperpigmentation at baseline. Relapse of melasma after treatment may also have been reduced due to a decrease in activated melanocytes (the DC count) [12]. This may suggest that OCT examination is sensitive for monitoring post-treatment cellular changes in melasma before the appearance of notable treatment efficacy. Consequently, microstructural changes in melasma may be observed in OCT earlier than the clinical improvement scored by the MASI, which has been used for a long time but may lag behind OCT changes. Nevertheless, we cannot assume these OCT changes will translate into durable clinical benefit without longer follow-up and larger case numbers. Whether OCT changes predict delayed clinical improvement or relapse is still uncertain currently.

**Figure 9:**
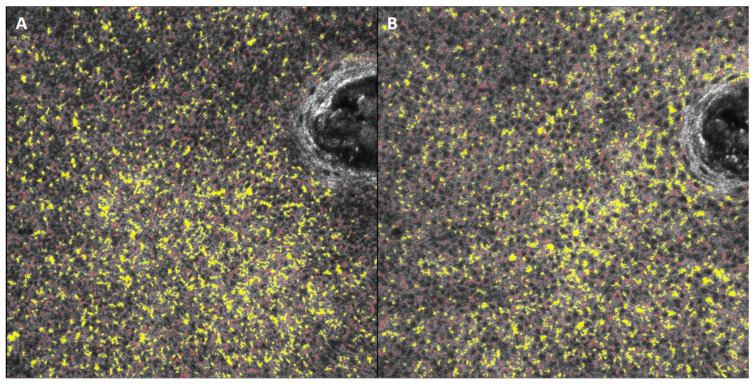
Optical coherence tomography (OCT) images of Patient 1 captured at a dermo-epidermal junction depth of 74 µm (**A**) and at a depth of 64 µm (10 µm above the dermo-epidermal junction) (**B**) with the confetti (yellow)/granular (red) melanin ratio being 249.85% (**A**) and 192.46% (**B**), respectively.

“Confetti melanin” refers to dense, large clusters and high-pigment packages in the basal layer, associated with hyperactive melanocytes [23]. Granular melanin, often located in treated melasma, generally represents smaller, dispersed pigment granules [23]. Consequently, higher levels of granular melanin may indicate successful, stable treatment compared to high levels of confetti melanin [23]. OCT is increasingly used to identify melanin types to evaluate treatment efficacy. Effective treatments often reveal a reduction in large “confetti” particles and an increase in, or maintenance of, finer “granular” melanins [23]. We further identified the determining factors for a good therapeutic response to Sylfirm X MRF. A confetti/granular melanin ratio ≥196% may demonstrate that the treatment effect of melasma remains the same or deteriorates compared to our results. This study makes a novel contribution to the filed via the characterization of responders and non-responders to MRF by OCT and the validation of the role of the confetti/granular melanin ratio. Larger melanin aggregates correspond to membrane-bound melanosome-rich packages [29,30,31,32]. Previous studies have reported reduced autophagy activity in darker skin-derived keratinocytes and impaired autophagy-related processes in melasma-affected skin [29,30,31,32]. In addition, melanosome degradation involves lysosomal and protease-related processing pathways within keratinocytes [29,30,31,32]. Therefore, larger, more aggregated melanosome-rich structures may exhibit altered degradation and clearance dynamics compared with finer granular melanin distributions [29,30,31,32]. The OCT images of the poor-response group (Patient 1 as a representative) captured at different depths are shown in Figure 9. The signals of confetti melanins (yellow) are more intense than those of granular melanins (red) and are more concentrated at the dermo-epidermal junction than the suprabasal layer.

The changes in epidermal thickness were paradoxical in this study; however, skin generally regenerates over a 27-day cycle, and epidermal thickness varies throughout the day due to factors such as hydration levels, body temperature, occlusion, mechanical stress and external environmental factors [33,34,35]. While the epidermis is relatively stable, it can show temporary daily fluctuations in thickness based on hydration status and pressure. In addition, the difference in epidermal thickness after microneedling RF treatments is not sufficiently integrated into the interpretation of results due to the small sample size [33,34,35].

This study has several limitations. The fact that we had a small sample size without a control group may prevent the findings from being extrapolated; thus, more randomized controlled trials with larger sample sizes are needed to validate these findings. Certain potential confounding factors, such as post-therapeutic topical corticosteroids which can reduce inflammation, were not excluded. The fair response classification may have partly reflected baseline severity rather than intrinsic biological responsiveness. Patient-reported outcomes were not presented due to the poor correlations between the objective severity scores and patients’ subjective perceptions of improvement. In addition, the resolution and maximum imaging depth achievable with OCT were relatively restricted compared to those of histological examinations. Consequently, the pathophysiology of melasma cannot be fully understood by using OCT alone. Mast cells, which release tryptase to induce basal cell membrane disruption and solar elastosis, phenomena associated with melasma, were not observed in the present study. Finally, the follow-up duration was short in our study given that melasma is prone to recurring. However, the present study showed that OCT-guided individualized therapy has a potential role in the treatment of melasma.

## 5. Conclusions

This is a pioneering study that has used OCT to assess the therapeutic efficacy of MRF in melasma and investigated the possible determining factors for good therapeutic response. MRF may be a treatment option for melasma in certain populations. The statistical results were exploratory and descriptive, and readers should avoid overinterpretation. Randomized, controlled, large-scale trials with OCT assessment should be conducted to validate that OCT may serve as a real-time, noninvasive, and in vivo tool for sequentially monitoring the evolution of melasma under treatment and investigating the underlying pathomechanisms, thereby guiding individualized treatment.

## Figures and Tables

**Table 1:** Baseline characteristics and comparison between characteristics of patients with fair and poor therapeutic responses.

	Total, *n* (%)	Fair Response, *n* (%)	Poor Response, *n* (%)	*p*-Value
Female, *n* (%)	4 (80)	2 (100)	2 (66.7)	
Age, (mean ± SD)	50.4 ± 4.6	47.5 ± 3.5	52.3 ± 4.7	0.31
Number of treatment, (mean ± SD)	3.8 ± 0.4	4.0 ± 0	3.7 ± 0.6	0.50
MASI score at baseline, (mean ± SD)	8.0 ± 2.7	10.2 ± 1.8	6.5 ± 2.1	0.04
MASI score at the last follow up, (mean ± SD)	5.7 ± 1.8	5.1 ± 0.3	6.1 ± 2.2	0.43

MASI, melasma area and severity index; SD, standard deviation.

## Data Availability

The data that support the findings of this study are available from the corresponding author upon reasonable request.

## Supplementary Materials

The following supporting information can be downloaded at: https://www.mdpi.com/article/10.3390/diagnostics16131957/s1, Figure S1: Representative example with artificial intelligence (AI)-generated overlays and corresponding clinician review. Table S1: The individual change of Melasma Area and Severity Index (MASI). Table S2: The individual change of basement membrane disruption and epidermal thickness. Figure S2: Individual patient’s trajectories for dendritic cell count and basement membrane disruption.
