# Supplementary material for: Retrospective Analysis of the Therapeutic Outcomes of Microneedle Radiofrequency on Melasma by Optical Coherence Tomography: A Observational Pilot Study
Source: Diagnostics (Basel). 2026 Jun 24;16(13):1957. doi: 10.3390/diagnostics16131957 (PMC13359605; doi:10.3390/diagnostics16131957)
Supplement: Supplementary file 1 [file diagnostics-16-01957-s001.zip › diagnostics-4228395-supplementary.pdf]

Supplementary material

Supplementary Figure S1. Representative example: artificial intelligence (AI)-generated overlays and corresponding clinician review.

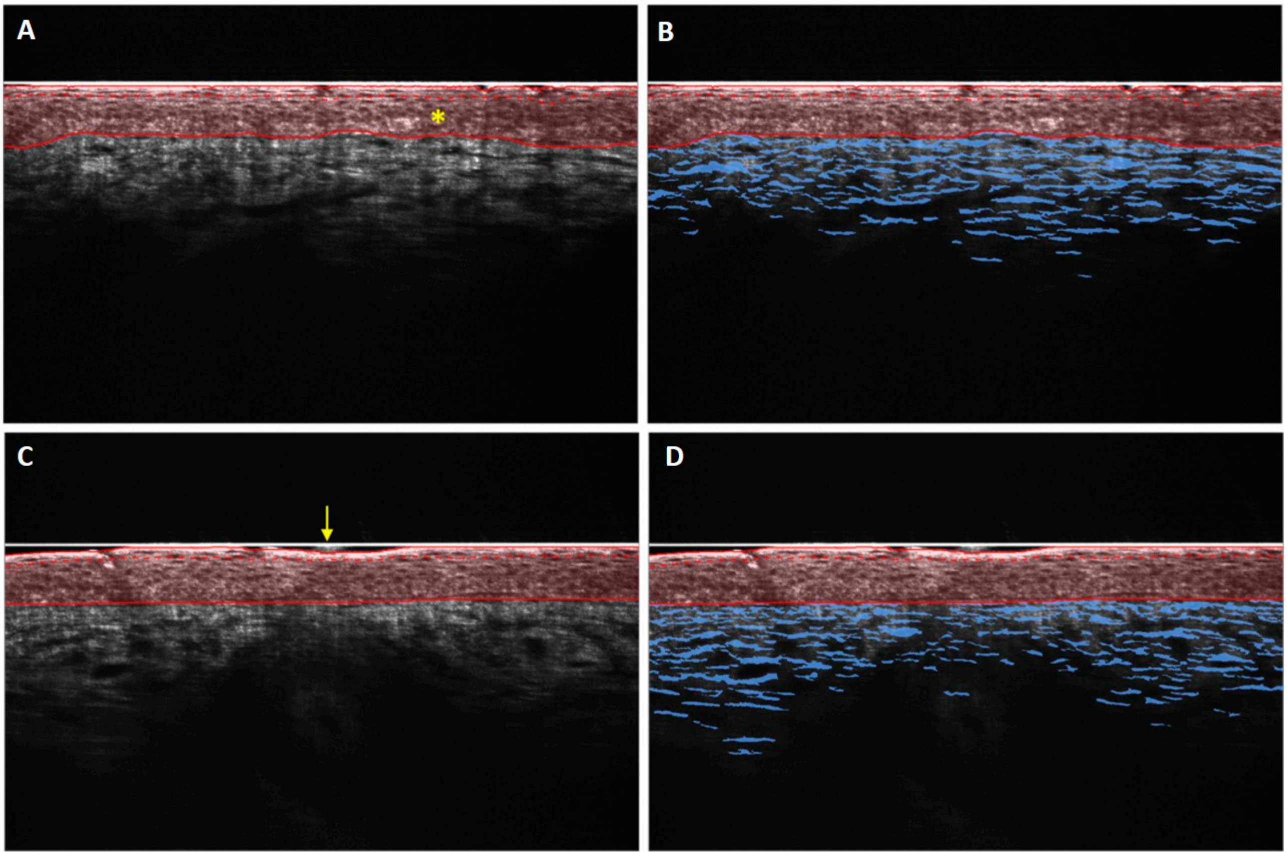

Collagen fiber detection in Patient 4 at baseline. (A) Original OCT image. The asterisk shows the melanin signal. (B) OCT image with AI-based collagen fiber detection overlay, demonstrating that collagen fiber segmentation remained detectable despite the presence of melanin signal. Clinician-reviewed results, included in the analysis. (C) Original OCT image. The arrow indicates the hair structure on the OCT image. This resulted in a reduced scattered-light signal in the upper dermis. Following clinician review based on predefined image-quality criteria, this image is excluded from AI analysis. (D) OCT image with AI-based collagen fiber detection overlay, demonstrating that the collagen fiber segmentation signal was reduced in the upper dermis.

Supplementary Table S1. Individual changes in The Melasma Area and Severity Index (MASI).

Patient	Doctor's assessment	Baseline MASI	1st MASI	2nd MASI	3rd MASI	4th MASI	Difference between baseline and last MASI	% MASI improvement
01	A	8.7	7.5	6.6	7.5	7.5	1.2	13.8%
	B	8.7	7.5	5.1	5.1	7.5	1.2	13.8%
02	A	7.8	7.8	7.8	-	-	0	0%
	B	6.6	7.8	7.8	-	-	1.2	18.2%
03	A	8.4	7.2	6.3	6.3	6.3	2.1	25%
	B	8.4	6.3	5.4	5.4	4.5	3.9	46.4%
04	A	3.6	2.4	2.4	3	3	0.6	16.7%
	B	3.6	2.4	2.4	3.6	3	0.6	16.7%
05	A	12	10.8	8.4	7.2	5.4	6.6	55%
	B	11.7	7.5	6.3	6.6	3.9	7.8	66.7%

MASI, Melasma Area and Severity Index.

Supplementary Table S2. Individual changes in basement membrane disruption and epidermal thickness.

Response group	Patient	BM disruption (µm), baseline	BM disruption (µm), after Tx
Poor response group	Patient 1	170.17±36.91	54.25±25.32
	Patient 2	30.00±32.32	0.00±0.00
	Patient 4	186.00±60.01	175.50±72.83
Fair response group	Patient 3	413.75±67.73	288.50±38.89
	Patient 5	401.33±83.93	232.00±96.17
**Response group**	**Patient**	**Epidermal thickness (µm), baseline**	**Epidermal thickness (µm), after Tx**
Poor response group	Patient 1	33.35±3.26	49.20±0.16
	Patient 2	43.15±3.20	42.09±1.97
	Patient 4	35.86±5.08	44.34±3.05
Fair response group	Patient 3	66.79±3.21	55.52±4.23
	Patient 5	57.47±2.66	50.94±0.21

BM, basement membrane; Tx, treatment.

Supplementary Figure S2. Individual patients' trajectories of dendritic cell count and basement membrane disruption.

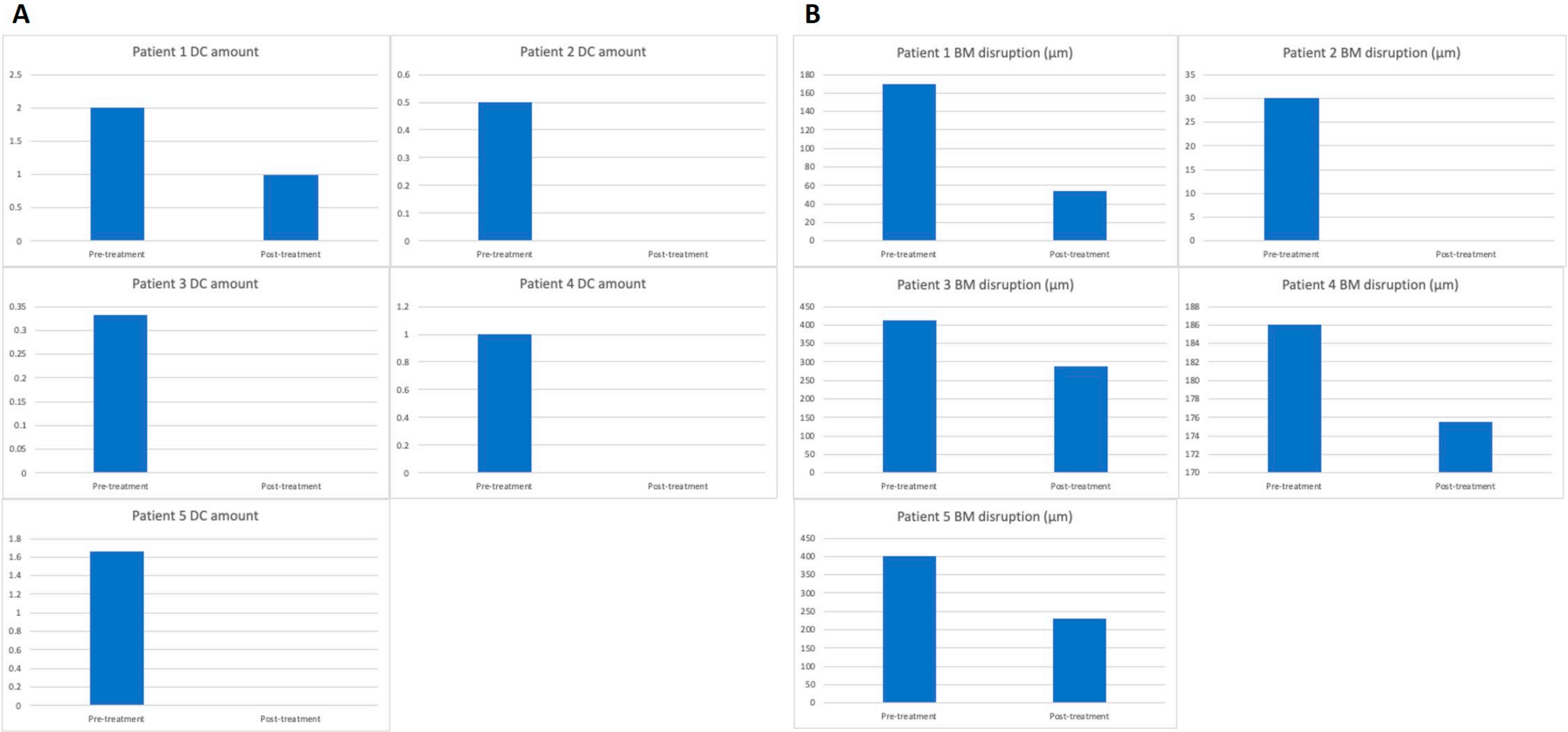

A decrease from pre-treatment to post-treatment in the dendritic cell count and basement membrane disruption was observed across all patients.

BM, basement membrane; DC, dendritic cell.
